# Narrow-band hard-x-ray lasing with highly charged ions

**DOI:** 10.1038/s41598-020-65477-0

**Published:** 2020-06-10

**Authors:** Chunhai Lyu, Stefano M. Cavaletto, Christoph H. Keitel, Zoltán Harman

**Affiliations:** Max-Planck-Institut für Kernphysik, Saupfercheckweg 1, 69117 Heidelberg, Germany

**Keywords:** Free-electron lasers, X-rays, Atomic and molecular interactions with photons

## Abstract

A scheme is put forward to generate fully coherent x-ray lasers based on population inversion in highly charged ions, created by fast inner-shell photoionization using broadband x-ray free-electron-laser (XFEL) pulses in a laser-produced plasma. Numerical simulations based on the Maxwell–Bloch theory show that one can obtain high-intensity, femtosecond x-ray pulses of relative bandwidths Δ*ω*/*ω* = 10^−5^–10^−7^, by orders of magnitude narrower than in x-ray free-electron-laser pulses for discrete wavelengths down to the sub-ångström regime. Such x-ray lasers can be applicable in the study of x-ray quantum optics and metrology, investigating nonlinear interactions between x-rays and matter, or in high-precision spectroscopy studies in laboratory astrophysics.

## Introduction

Most x-ray free-electron laser (XFEL) facilities in operation or under construction generate hard-x-ray pulses based on the self-amplified spontaneous-emission (SASE) process. Despite their broad (Δ*ω*/*ω* ~ 10^−3^) and chaotic spectrum^1^, such high-intensity, transversely coherent SASE x-ray pulses have found diverse applications in physics, chemistry and biology^2–4^. However, current and future investigations of x-ray quantum optics^5–7^, nonlinear x-ray scattering^8^, high-resolution x-ray spectroscopy^9,10^, frequency-resolved x-ray scattering^11,12^ and coherent x-ray pump-probe experiments^13–15^ also require a longitudinally coherent x-ray source with a narrower and smooth spectrum. Therefore, considerable efforts have been devoted to generate x-ray pulses with better temporal coherence and lower bandwidth^5,16–23^.

Different seeding schemes have been implemented successfully at XFEL facilities^16–20^. In the hard-x-ray regime, the self-seeding mechanism has reduced the relative bandwidth to the level of 5 × 10^−5^ at photon energies of 8–9 keV (ref. ^17^). However, at higher energies around 30 keV, the predicted relative bandwidth for seeded XFELs is approximately 4 × 10^−4^ (ref. ^1^). Further reduction of the bandwidth with low-gain XFEL oscillators (XFELOs) has also been proposed. By recirculating the x-ray pulses through an undulator in a cavity, the output x-rays have an estimated relative bandwidth as small as 10^−7^ (refs. ^5,21^). To date, however, the XFELO scheme remains untested.

X-ray lasers (XRLs) adopting atomic transitions provide an alternative approach towards x-ray sources with high brightness and temporal coherence^22–28^. Plasma-based XRLs have achieved saturated amplification for different wavelengths in the soft-x-ray regime. Such soft-XRLs are mainly based on the 3*p* → 3*s* or 4*d* → 4*p* transitions in Ne- or Ni-like highly charged ions (HCIs) for elements varying from Si to Au, where the population inversion is achieved through electron collisional excitation in a hot dense plasma with lengths from a few centimeters up to tens of centimeters^26–28^. By employing a seeding or a different pumping scheme^28^, saturated soft-x-ray lasing from a millimeter-long high-gain plasma has also been realized. The first hard-XRL was initially proposed through direct pumping of the 1*s*^−1^ → 2*p*^−1^ transition via photoionization of a K-shell electron^29^. Limited by the high pump power required, this scheme was demonstrated only in recent years after XFELs became available. As a result of the high XFEL-pumping efficiency, an XRL at 846 eV with saturated intensity was achieved in a 2.8-mm-long neon gas^22^, with an estimated fractional bandwidth of 3 × 10^−4^ due to fast Auger decay of the upper lasing state. In an experiment with solid copper, an XRL with a sub-saturation intensity at a photon energy of 8 keV was produced^23^, with a measured relative bandwidth of 2 × 10^−4^. Reduction of the bandwidth by another order of magnitude via the long-lived 2*p* → 1*s* dipole transition in Ne^9+^ generated by the XFEL pump pulse was also discussed^30^, but it becomes inefficient for heavier ions with transition energies larger than 10 keV. For example, the spontaneous emission rate of the 2*p* → 1*s* electric-dipole transition, scaling as *Z*^4^ (with *Z* the atomic number), is around 1/160 fs^−1^ for Ne^9+^ with a transition energy of 1.022 keV, and becomes 1/1.7 fs^−1^ for Ga^30+^ with a transition energy of 10 keV. Alternative schemes to obtain narrow-band XRLs at even higher photon energies, e.g., deep into the hard-x-ray regime, are thus required.

**Figure 1:**
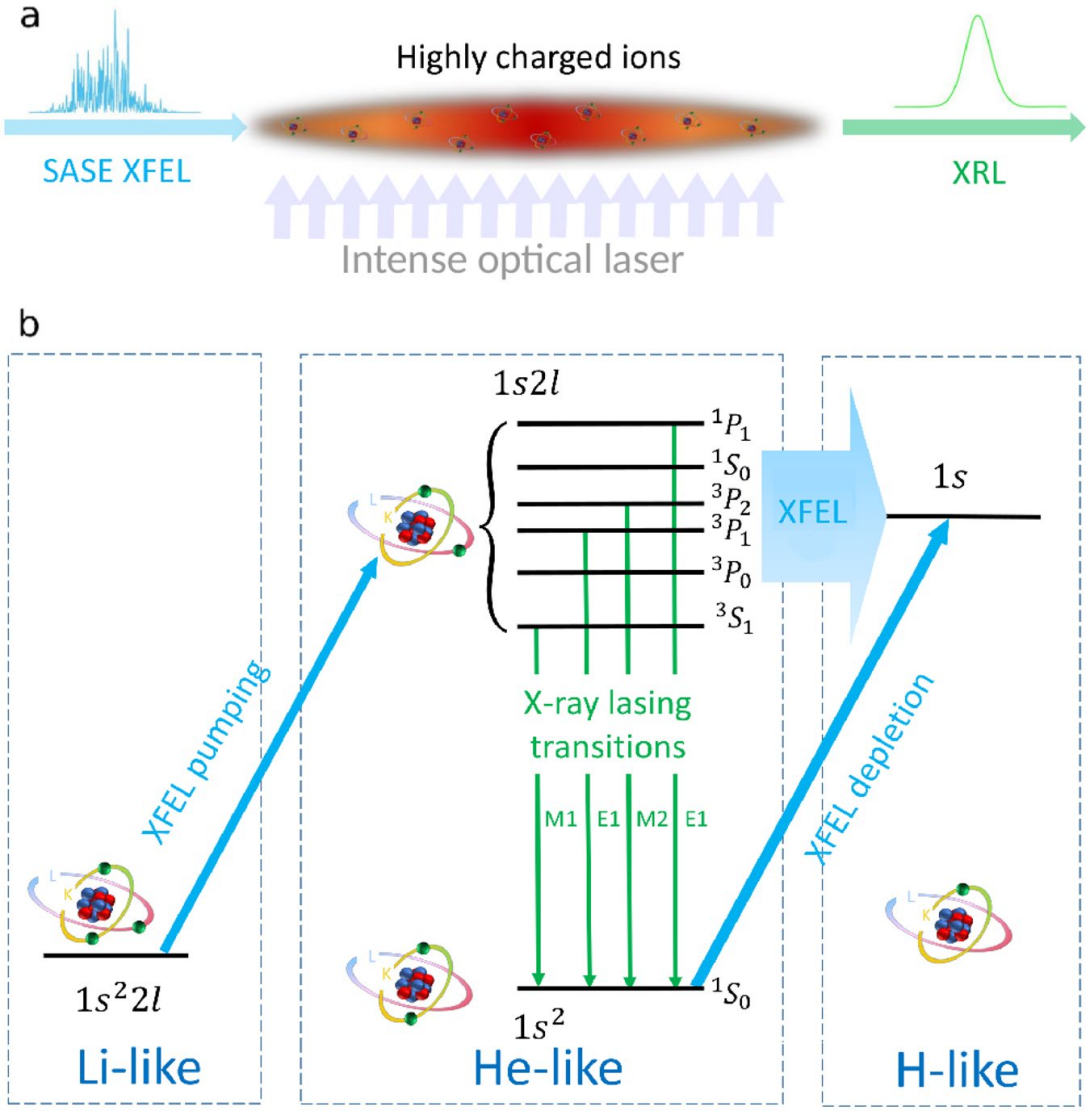
Scheme of the lasing process. (**a**) Laser configuration. (**b**) Level scheme. The order of the 1*s*2*l* (*l* = *s*, *p*) states may vary for different elements (see Table 1). Figure adapted from C. Lyu’s Ph.D. thesis^99^.

Here we put forward a plasma-based XRL based on the 1*s*2*l* → 1*s*^2^ (*l* = *s*, *p*) transition in He-like HCIs and pumped by an XFEL pulse (Fig. 1). The detrimental Auger-decay channel is nonexistent due to the lack of outer-shell electrons. By choosing a non-dipole lasing transition which decays slowly, this enables saturated lasing for photon energies well above 10 keV and it results in a further reduction of the XRL bandwidth by several orders of magnitude. Such coherent XRLs would enable applications in the growing field of x-ray quantum optics^5–7,31–37^ and metrology^9,10^, in addition to investigations of nonlinear interactions between x-rays and matter^8,38–44^, and high-precision spectroscopy studies in laboratory astrophysics^45,46^.

## Results

### X-ray lasing scheme

The photoionization-pumped atomic laser is illustrated in Fig. 1a. First, a line-focused intense optical laser generates a plasma of highly charged ions, which mainly consists of Li-like ions in a 1*s*^2^2*l* state and He-like ions in a 1*s*^2^ state (see Methods). Then, a SASE XFEL pulse, tuned above the K-edge of He-like ions in the 1*s*^2^ state, is injected perpendicularly to the optical laser beam. This XFEL pulse immediately removes a K-shell electron from each of the Li- and He-like ions, creating He-like ions in the 1*s*2*l* excited states and H-like ions in the 1*s* state, respectively (blue arrows in Fig. 1b). Further K-shell ionization from these states is prevented by higher binding energies. Subsequent decay of He-like ions from the 1*s*2*l* state to the 1*s*^2^ ground state (green arrows in Fig. 1b) leads to emission of x-ray photons via four possible K_*α*_ transitions: one magnetic-dipole (*M*1) transition from the ^3^*S*_1_ state, two electric-dipole (*E*1) transitions from the ^3^*P*_1_ or ^1^*P*_1_ states, and one magnetic-quadrupole (*M*2) transition from the ^3^*P*_2_ state. The energy and lifetime of these transitions for suitable XRL systems are shown in Table 1, while Table 2 displays K- and L-shell ionization thresholds^47^. The ionization-potential depression in the plasma is small and can thus be neglected^48^. The far-detuned L-shell photoionization (broad blue arrow in Fig. 1b) may also depopulate the excited state, but its rate is much slower compared to the pump rates. Thus, the slow decay of these excited states leads to a transient population inversion in the He-like ions, and to the amplification of the emitted x-rays, i.e., transient x-ray lasing.

**Table 1:** Energy $\hslash {\omega }_{0}$ and lifetime *τ* of the 1*s*2*l* states in He-like ions.

1*s*2*l*	Ne^8+^		Ar^16+^		Kr^34+^		Xe^52+^	
	$\hslash {\omega }_{0}$	*τ*	$\hslash {\omega }_{0}$	*τ*	$\hslash {\omega }_{0}$	*τ*	$\hslash {\omega }_{0}$	*τ*
	(keV)	(s)	(keV)	(s)	(keV)	(s)	(keV)	(s)
^1^*P*_1_:* E*1	0.9198	1.08 × 10^−13^	3.137	9.11 × 10^−15^	13.111	6.53 × 10^−16^	30.625	1.47 × 10^−16^
^1^*S*_0_: ×	0.9134	3.14 × 10^−04^	3.122	6.33 × 10^−06^	13.024	1.26 × 10^−06^	30.210	1.66 × 10^−07^
^3^*P*_2_:* M*2	0.9125	6.24 × 10^−09^	3.124	1.24 × 10^−09^	13.087	9.46 × 10^−12^	30.589	3.44 × 10^−13^
^3^*P*_1_:* E*1	0.9123	1.90 × 10^−10^	3.121	5.67 × 10^−13^	13.023	2.54 × 10^−15^	30.201	3.27 × 10^−16^
^3^*P*_0_: ×	0.9122	6.77 × 10^−09^	3.120	3.41 × 10^−09^	13.020	1.34 × 10^−09^	30.207	6.39 × 10^−10^
^3^*S*_1_:* M*1	0.9025	1.10 × 10^−04^	3.101	2.30 × 10^−07^	12.976	1.80 × 10^−10^	30.124	2.68 × 10^−12^

Values are obtained from multiconfiguration Dirac–Hartree–Fock calculations^55^. The K_*α*_ transitions from the ^1^*S*_0_ and ^3^*P*_0_ states in the 1*s*2*l* configuration to the ^1^*S*_0_ state in the 1*s*^2^ configuration are forbidden by selection rules, and their radiative decay to the other 1*s*2*l* states is negligibly slow as indicated by the long lifetime. The lifetimes of the lasing transitions, satisfying the condition 1 fs < *τ* < 10 ps, are underlined.

**Table 2:** K- and L-shell ionization thresholds $\hslash {\omega }_{{\rm{T0}}}$, $\hslash {\omega }_{{\rm{Tg}}}$, $\hslash {\omega }_{{\rm{Te}}}$, $\hslash {\omega }_{{\rm{T2}}}$ and cross sections *σ*_0_, *σ*_g_, *σ*_e_.

1*s2l*	1*s*^2^2*l* → 1*s*2*l*		1*s*^2^ → 1*s*		1*s*2*l* → 1*s*		1*s*2*l* → 2*l*
	$\hslash {\omega }_{{\rm{T0}}}$	*σ*_0_	$\hslash {\omega }_{{\rm{Tg}}}$	*σ*_g_	$\hslash {\omega }_{{\rm{Te}}}$	*σ*_e_	$\hslash {\omega }_{{\rm{T2}}}$
	(keV)	(kb)	(keV)	(kb)	(keV)	(kb)	(keV)
Ne^8+ 1^*P*_1_	1.138	39.1	1.196	151	0.274	1.55	1.295
Ar^16+ 3^*P*_1_	4.013	24.6	4.124	44.6	0.998	0.48	4.312
Ar^16+ 1^*P*_1_	4.029	12.4	4.124	44.6	0.982	0.47	4.312
Kr^34+ 3^*P*_2_	17.048	6.24	17.315	10.9	4.206	0.12	17.635
Xe^52+ 3^*P*_2_	39.949	2.66	40.302	5.31	9.671	0.06	40.961

The photoionization cross sections *σ*_0_, *σ*_g_ and *σ*_e_ (kb = 10^−21^ cm^2^) are calculated with the LANL Atomic Physics Codes^47^, by assuming a corresponding XFEL photon energy of *ω*_xfel_ = *ω*_Tg_. The cross sections *σ*_0_ and *σ*_g_ only account for the ionization channel to and from the corresponding upper lasing state in the first column, respectively. Values for other channels can be found in the Supplemental Materials. Due to ${\omega }_{{\rm{T2}}}-{\omega }_{{\rm{Tg}}}\gg \Delta \,{\omega }_{{\rm{xfel}}}$ (Δ*ω*_xfel_ is the bandwidth of the corresponding XFEL pulse), the single-photon ionization channel 1*s*2*l* → 2*l* is energetically forbidden.

### Lasing transitions

Two factors determine which transition will lase. First, typical XFEL facilities operate at peak photon fluxes of 10^33^–10^35^ ph./cm^2^/s (refs. ^1,4^). For the photoionization cross sections shown in Table 2 (ref. ^47^), they yield inverse ionization rates of a few femtoseconds. Transitions with lifetimes longer than 1 fs are thus necessary to ensure population inversion. Second, for the ions we are going to consider, the hydrodynamic plasma expansion time of ~10 ps (refs. ^49,50^), depending on the plasma size and temperature, also influences the lasing process. Sufficient x-ray amplification takes place only from transitions whose upper-state lifetimes satisfy 1 fs < *τ* < 10 ps. Systems and transitions satisfying this condition are underlined in Table 1. Although current XFEL facilities are designed to deliver x-rays with a single-photon energy up to 30 keV (ref. ^1^), calculations for Xe^52+^ requiring 40.3-keV FEL frequencies were included as an example of the applications which would be enabled by such XFEL pulses in the near future^4^.

For light ions, Ne^8+^ for example, the *E*1 transition with a decay rate of 9.2 × 10^12^ s^−1^ from the ^1^*P*_1_ state will develop lasing. The other transitions have decay times much larger than the plasma expansion time, and their contribution is negligible compared to the ^1^*P*_1_ state. For heavy ions such as Xe^52+^, however, the two *E*1 transition rates scale as ~*Z*^4^ (*Z* being the atomic number), corresponding to 3.0 × 10^15^ s^−1^ for ^3^*P*_1_ and 6.8 × 10^15^ s^−1^ for ^1^*P*_1_, respectively, which are too large to enable population inversion with available XFEL pulses. On the other hand, the decay rates of the *M*1 transition from the ^3^*S*_1_ state and of the *M*2 transition from the ^3^*P*_2_ state are 3.7 × 10^11^ s^−1^ and 2.6 × 10^12^ s^−1^, respectively, which is sufficient for lasing to take place before the expansion of the plasma.

### Two-level approximation

Since the lifetimes of different K_*α*_ transitions from the 1*s*2*l* configuration differ by orders of magnitude, when we model the lasing process from a specific transition, a two-level approximation for the He-like ions is applicable. This is evident for the case of Ne^8+^ ions, where only the *E*1 transition from the ^1^*P*_1_ state satisfies the requirements for lasing described above. For Ar^16+^ ions, both *E*1 transitions may lase simultaneously. Thus, the XFEL photon energy *ω*_xfel_ and the mean peak flux have to be tuned properly, such that when the transition from the ^3^*P*_1_ state is pumped, the other transition does not lase due to the relatively fast decay rate of the ^1^*P*_1_ state. On the other hand, when lasing from the ^1^*P*_1_ state is considered, the effects from the ^3^*P*_1_ state can be neglected as well due to its slow lasing dynamics. Similar approximations can be applied when we model the XRLs from Kr^34+^ and Xe^52+^ ions which also contain two suitable lasing transitions. Furthermore, for the plasma conditions considered later, collision-induced population transfer rates between different upper states are small compared to the K_*α*_ emission rate, ensuring the validity of the two-level approximation.

### Initial charge-state distributions

**Figure 2:**
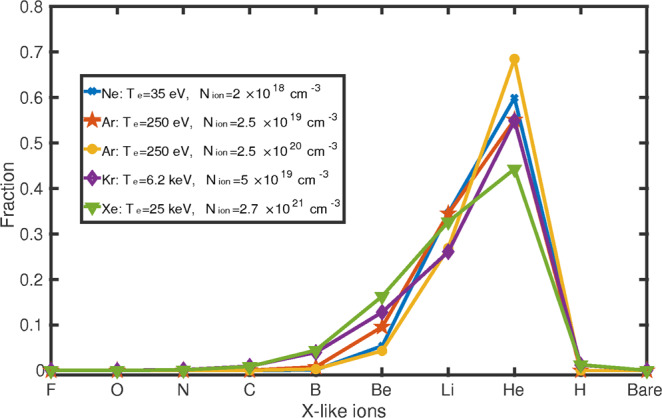
Charge-state distributions in laser-produced plasmas. Electron temperature *T*_e_ and ion density *N*_ion_ are chosen to enable a significant fraction of Li-like ions in the plasma^51^. For the Ar plasma, the red (yellow) line with *N*_ion_ = 2.5 × 10^19^ cm^−3^ (*N*_ion_ = 2.5 × 10^20^ cm^−3^) is tuned to achieve optimal lasing from the ^3^*P*_1_ (^1^*P*_1_) state. Figure reproduced from C. Lyu’s Ph.D. thesis^99^.

Our scheme requires a plasma in which the density of Li-like ions is maximized compared to other ionic species. While the generation of such a plasma, in the initial stage (of a few femtoseconds), involves highly complex and nonlinear laser–matter interaction processes for the ejection of outermost electrons, the removal of deeply bounded electrons in the later stage of the plasma generation is dominated by linear processes such as collisional ionization. Therefore, when we focus on the state of the plasma after the HCIs are generated, collisional–radiative atomic-physics plasma codes such as FLYCHK^51^ can be used for an estimation of the properties of the plasma, such as the ion density *N*_ion_ and the electron temperature *T*_e_ necessary to satisfy the corresponding ionic distributions. Then, the pondermotive scaling law can be used to estimate the laser intensity and wavelength needed to produce such a plasma (see Discussions). The charge-state distributions obtained from such simulations are displayed in Fig. 2, where ions with other charge states coexist with the Li-like ions. We notice in particular that most of the ions are He-like ions in the 1*s*^2^ state with negligible populations in the 1*s*2*l* state. This is due to the fact that, even though energetic electrons can impact with the ions of any charge state and create K-shell holes, these are immediately filled by the outer-shell electrons, leading to the emission of incoherent K_α_ photons in the hard-x-ray regime^52–54^.

Electron-impact K-shell ionization or excitation are very slow compared to the spontaneous decay of the excited state, such that population inversion does not set in and spontaneous K_α_ radiation is not amplified. However, when an intense XFEL pulse is injected, the K-shell photoionization rates of the Li-like ions can be much larger than the decay rate of the 1*s*2*l* excited state of the resulting He-like ions. Since the lower lasing state (1*s*^2^) of the He-like ions is also depleted by the XFEL pulse with a large photoionization rate, population inversion in the He-like ions will be reached shortly after the arrival of the XFEL pulse.

### XRLs from He-like Ne and Ar

Parameters for x-ray lasing from transitions in He-like Ne and Ar ions are shown in Table 3. The natural linewidth Γ = 1/*τ* for each lasing transition is obtained from multiconfiguration Dirac–Hartree–Fock calculations^55^. Doppler broadening Δ*ω*_D_, electron–ion impact broadening Δ*ω*_e−i_, and ion–ion Stark broadening Δ*ω*_i−i_, are calculated for given *T*_e_ and *N*_ion_ as shown in Fig. 2 based on Maxwell–Boltzmann distributions^56^, with ion temperature *T*_i_ = 500 K (see Methods). XFEL parameters such as peak flux and pulse duration Δ*t*_xfel_ ≈ 0.2*τ* are selected in order to ensure photoionization rates larger than the decay rate of the 1*s*2*l* excited state in He-like ions, and the onset of population inversion. The XFEL spectral width Δ*ω*_xfel_ is set by assuming an XFEL coherence time of 0.01*τ* for the sake of computational cost. Though such an assumption may result in $\Delta {\omega }_{{\rm{xfel}}}/{\omega }_{{\rm{xfel}}}\ll \mathrm{0.5 \% }$, see, e.g., Table 4 for Kr and Xe ions, it does not modify the pumping efficiency as the photoionization cross section varies slowly within the pulse bandwidth, which is 1% of the central photon energy. The focal size *S*_focal_ of the XFEL pulse is chosen to ensure the required peak flux for the x-ray photon energy listed in Table 3, as well as to ensure that the divergence of the XFEL beam is negligible during its propagation through the plasma (see Methods).

**Table 3:** XRL transitions in He-like Ne and Ar ions.

Transitions			Line broadenings			XFELs				Simulation results		
Upper state	$\hslash {\omega }_{0}$	$\hslash \varGamma$	$\hslash \Delta {\omega }_{{\rm{D}}}$	$\hslash \Delta {\omega }_{{\rm{e}}-{\rm{i}}}$	$\hslash \Delta {\omega }_{{\rm{i}}-{\rm{i}}}$	peak flux	Δ*t*_xfel_	$\hslash \Delta {\omega }_{{\rm{xfel}}}$	*S*_focal_	*L*_c_	*I*_c_	Δ*ω*/*ω*
1*s*2*l*	(keV)	(meV)	(meV)	(meV)	(meV)	(ph./cm^2^/s)	(fs)	(eV)	(*μ*m^2^)	(mm)	(W/cm^2^)	
Ne^8+ 1^*P*_1_	0.920	6.08	3.23	0.08	0.06	1.1 × 10^34^	21.3	6.25	1.5	2.8	${4.5}_{-4.4}^{+9.3}\times {10}^{12}$	${7.8}_{-2.7}^{+4.3}\times {10}^{-5}$
Ar^16+ 3^*P*_1_	3.121	1.16	7.80	1.38	9.81	2.3 × 10^33^	124	1.55	1.0	3.3	${5.0}_{-4.0}^{+3.8}\times {10}^{14}$	${7.8}_{-2.1}^{+1.9}\times {10}^{-6}$
Ar^16+ 1^*P*_1_	3.137	72.2	7.83	2.20	25.22	2.0 × 10^35^	2.0	74	0.1	0.35	${1.5}_{-0.8}^{+1.4}\times {10}^{16}$	${4.7}_{-1.7}^{+2.0}\times {10}^{-5}$

**Figure 3:**
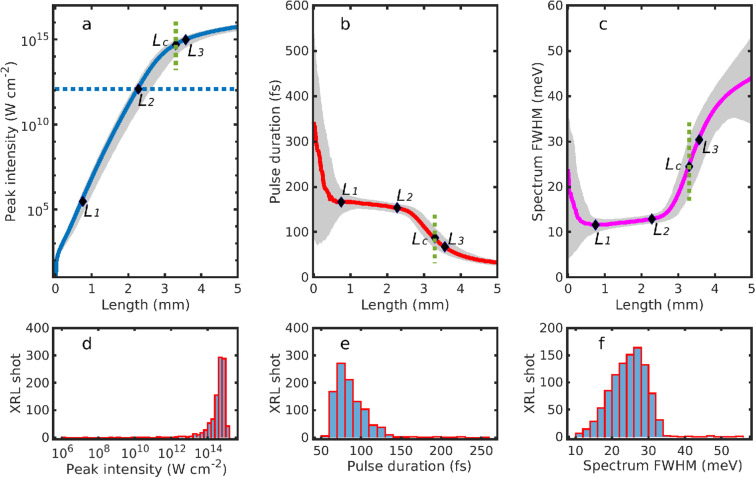
Evolution of the XRLs over 1,000 simulations (Ar^16+ 3^*P*_1_). (**a**–**c**) Peak intensity, pulse duration and spectral full width at half maximum (FWHM) for the x-ray laser. The solid lines display results averaged over 1,000 simulations. The dotted line in (**a**) indicates the saturation intensity *I*_s_ = 1.18 × 10^12^ W cm^−2^. *L*_1_, *L*_2_ and *L*_3_ mark the lengths at which the XRL pulse reaches transform-limited profile, saturation intensity and Rabi flopping, respectively. *L*_c_ refers to the characteristic length at which the slope of the solid line in (**a**) is 1/3 of the slope at *L*_2_. The gray areas in (**a**–**c**) indicate the distribution areas of the results over 1,000 simulations. At a given length, the bottom and top edges of the areas indicate the 10th and 90th percentiles of the distributions, respectively. (**d**–**f**) Distributions of the peak intensity, pulse duration and spectral FWHM at *L*_c_ along the green dotted lines in (**a**–**c**). Figure reproduced from C. Lyu’s Ph.D. thesis^99^.

For the XFEL peak flux, ph. stands for photons. The uncertainties in the simulation results for *I*_c_ and Δ*ω*/*ω* arise from the random XFEL pulse profiles and the noisy spontaneous-emission seedings, with the upper and lower bounds being the values at the 10th and 90th percentiles of the corresponding distributions [as shown in Fig. 3(d,f) for Ar^16+ 3^*P*_1_]. Table adapted from C. Lyu’s Ph.D. thesis^99^.

**Table 4:** Same as Table 3 for He-like Kr and Xe ions.

Transitions			Line broadenings			XFELs				Simulation results		
Upper state	$\hslash {\omega }_{0}$	$\hslash \varGamma$	$\hslash \Delta {\omega }_{{\rm{D}}}$	$\hslash \Delta {\omega }_{{\rm{e}}-{\rm{i}}}$	$\hslash \Delta {\omega }_{{\rm{i}}-{\rm{i}}}$	peak flux	Δ*t*_xfel_	$\hslash \Delta {\omega }_{{\rm{xfel}}}$	*S*_focal_	*L*_c_	*I*_c_	Δ*ω*/*ω*
1*s*2*l*	(keV)	(meV)	(meV)	(meV)	(meV)	(ph./cm^2^/s)	(fs)	(eV)	(*μ*m^2^)	(mm)	(W/cm^2^)	
Kr^34+ 3^*P*_2_	13.087	0.07	22.58	0.45	2.76	1.9 × 10^34^	202	0.57	0.10	289	${4.3}_{-4.2}^{+7.6}\times {10}^{16}$	${3.7}_{-1.1}^{+1.6}\times {10}^{-7}$
Xe^52+ 3^*P*_2_	30.589	1.91	42.16	7.60	126	7.4 × 10^34^	72.5	1.96	0.13	8.5	${4.7}_{-4.5}^{+6.9}\times {10}^{18}$	${1.5}_{-0.5}^{+0.6}\times {10}^{-6}$

The values of the bandwidths Δ*ω*_xfel_ are relatively smaller than the realistic values at XFEL facilities (see Methods), and are used for the sake of computational cost. Using realistic bandwidths does not modify the conclusions as long as the photoionization cross section changes slowly within the realistic frequency bands. Therefore, when we compare the bandwidth of the XRLs and XFEL pulses, we refer to the realistic bandwidth measured at XFEL facilities and not to the values used in this table. Table adapted from C. Lyu’s Ph.D. thesis^99^.

We simulate the lasing process by solving the Maxwell–Bloch equations^57^ numerically in retarded-time coordinates for 1,000 different realizations of SASE XFEL pulses. The propagation and absorption of the XFEL pulses are accounted for through rate equations describing the evolution of the corresponding photon flux, while the XRL is modeled as an electromagnetic field in resonance with the corresponding lasing transition of the He-like ions. The results in Table 3 present the XRL intensity *I*_c_ and relative bandwidth Δ*ω*/*ω* at the characteristic plasma length *L*_c_, with *L*_c_ being the optimal length (as defined in Fig. 3) to obtain high-intensity x-ray pulses with narrow bandwidth. All the transitions are predicted to generate high-intensity x-ray pulses for plasma lengths around 3 mm or shorter, with a 2-order-of-magnitude improvement in Δ*ω*/*ω* compared to SASE XFEL pulses^1^ and XRLs with neutral atoms^22,23^.

### Evolution of the XRLs

**Figure 4:**
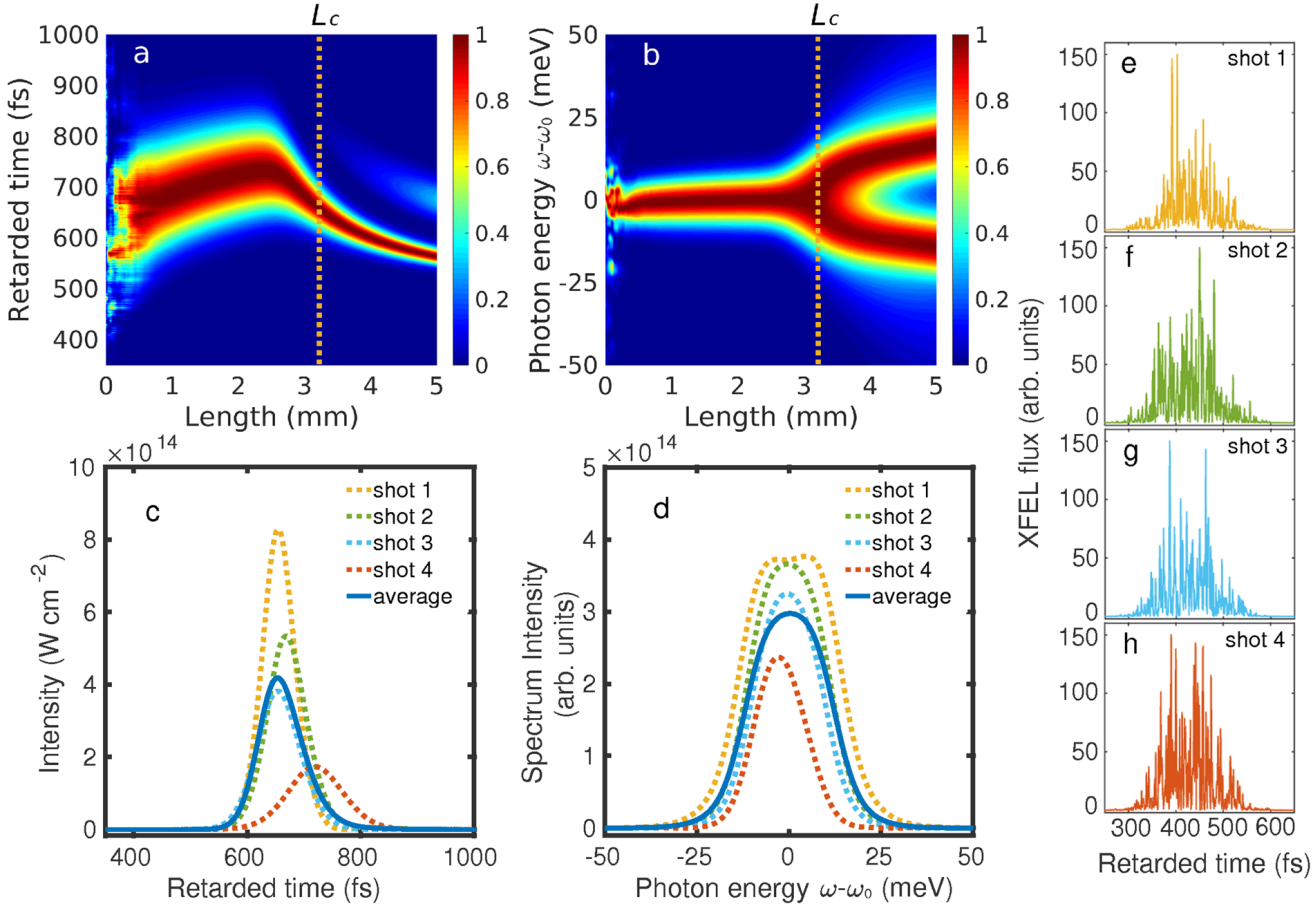
Pulse and spectrum profiles of a single-shot XRL (Ar^16+ 3^*P*_1_). (**a**,**b**) Evolution of normalized XRL intensity and power spectrum as a function of plasma length. Color bars: at each plasma length, the temporal and spectral intensities are normalized to their peak values at that length. The strength of the second peak appearing for the intensity profile at large plasma lengths in **a** has been multiplied by a factor of 5 for better visibility. (**c**,**d**) XRL pulse profile and spectrum at *L*_c_ for the different realizations of SASE XFEL pulses shown in (**e**–**h**). The yellow dotted lines, corresponding to the results from the simulation in (**a**,**b**), are associated with shot 1 in (**e**). The evolution of XRL intensity and spectrum for the XFEL shots 2–4 in (**f**–**h**), and averaged over 1,000 simulations (blue solid lines), can be found in the Supplemental Material. Figure reproduced from C. Lyu’s Ph.D. thesis^99^.

To understand the properties of our XRL and how it develops in the plasma, simulation results for the ^3^*P*_1_ → ^1^*S*_0_ transition in Ar^16+^ are shown in Figs. 3 and 4. We use a partial-coherence method^58^ to simulate 124-fs-long SASE XFEL pulses with a spectral width of $\hslash \Delta {\omega }_{{\rm{xfel}}}=1.55$ eV and peak photon flux of 2.3 × 10^33^ cm^−2^ s^−1^. This results in a peak pumping rate of 5.6 × 10^13^ s^−1^ for the upper lasing state, and a depletion rate of 1.0 × 10^14^ s^−1^ for the lower lasing state. They are 32 and 58 times larger than the spontaneous-emission rate of the ^3^*P*_1_ state, ensuring population inversion.

Averaged results over 1,000 SASE-pulse realizations are shown in Fig. 3a–c. The peak intensity of the XRL, shown by the solid line in Fig. 3a, increases exponentially during the initial propagation stage, then displays a saturation behavior. The dotted line indicates the saturation intensity ${I}_{{\rm{s}}}=\hslash \Gamma {\omega }_{0}^{3}/6\pi {c}^{2}$ at which the stimulated-emission rate equals the spontaneous-emission rate. This is also the intensity from which the amplification begins to slow down. The evolution of the pulse duration and the spectral width are shown by the solid lines in Fig. 3b,c. At *L* = 0, only spontaneous emission takes place: the 342-fs average pulse duration is mainly determined by the lifetime of the ^3^*P*_1_ state (Fig. 3b), whereas the 23.5-meV intrinsic spectral width before propagation (Fig. 3c) is mostly due to the sum of the natural linewidth Γ and the three broadening effects shown in Table 3.

During its propagation in the medium, gain narrowing and saturation rebroadening will also contribute to the final bandwidth. This can be observed by inspecting the four distinct propagation regions separated by *L*_1_, *L*_2_ and *L*_3_ in Fig. 3a–c, which can also be followed in Fig. 4a,b for a single simulation. Up to *L*_1_ = 0.75 mm, both the pulse duration and spectral FWHM decrease severely. The laser intensity and spectrum in this region for a single simulation are spiky and noisy, as the ions irradiate randomly in time and space (Fig. 4a,b). When the spontaneously emitted signal propagates and stimulated emission sets in, it selectively amplifies the frequencies around *ω*_0_ such that the XRL pulse approaches a fully coherent transform-limited profile at *L*_1_ (ref. ^59^), with a bandwidth smaller than the intrinsic width. Thereafter, a gradual broadening of the spectrum is observed in the region *L*_1_*L*_2_. The broadening increases abruptly from *L*_2_ = 2.3 mm, where the saturation intensity has been reached and the stimulated-emission rate exceeds the spontaneous-emission rate. This is accompanied by a substantial slowing down of the amplification of the intensity and a significant decrease of the pulse duration in the region between *L*_2_ and *L*_3_ (Fig. 3a,b). Further propagation of the XRL pulse after *L*_3_ = 3.5 mm is characterized by the onset of Rabi flopping (Fig. 4a) which is reflected by a splitting in the XRL spectrum (Fig. 4b). This effect is much stronger and more marked than for previous XFEL-pumped transient lasers with neutral atoms^59^ due to the absence of Auger decay^58^. At the same time, the gain of the laser intensity in this region is strongly suppressed.

### Optimal plasma length

The optimal choice for a coherent XRL pulse is located in the third region *L*_2_*L*_3_, where saturation has already been reached while the bandwidth is still narrow. By choosing the medium length to be *L*_c_ = 3.3 mm, as shown in Table 3 and Fig. 3, one will obtain an approximately 87-fs-long XRL pulse with an average peak intensity of *I*_c_ = 5.0 × 10^14^ W cm^−2^ (~80% fluctuations) and an average bandwidth of $\hslash \Delta \omega =24.5$ meV (~30% fluctuations). This can also be seen in Fig. 4(c,d) which show four single-shot XRL pulse and spectrum profiles at length *L*_c_ for the different realizations of SASE XFEL pulses. On average, the XRL from He-like Ar ions gives Δ*ω*/*ω* = 7.8 × 10^−6^ for a total of 8.7 × 10^8^ coherent photons, with a peak brightness of 8.1 × 10^30^ brt (brt = photons/s/mm^2^/mrad^2^/0.1%bandwidth is the units of the brightness). Feasibility of our narrowband lasing scheme with the inclusion of plasma inhomogeneities is discussed in the Supplemental Material.

### XRLs from He-like Kr and Xe

In the light of the promising results obtained for Ne and Ar, we use our model to predict XRL for more highly charged ions as well. For Kr^34+^ and Xe^52+^ ions, the *M*2 transitions provide an even more significant reduction of the bandwidth, with Δ*ω*/*ω* being 3 × 10^−7^ and 1.5 × 10^−6^, respectively. The resulting 13- and 30-keV lasers feature similar bandwidths as the untested XFELO scheme^21^, with intensities of ~10^18^ W cm^−2^. The relative bandwidths are by 2 to 3 orders of magnitude narrower than the value predicted for future seeded-XFEL sources at analogous hard-x-ray wavelengths around 0.41–0.95 Å (ref. ^1^). The experimental generation of such highly charged ions would be challenging^53^, albeit in principle possible via currently available petawatt laser facilities^60^.

Due to large broadening effects, the effective gain coefficient for the XRL from the *M*2 transition in Kr^34+^ ions is small, leading to an optimal plasma length of 29 cm. Though such a plasma can be generated with an intense optical laser, diffraction of the XFEL pump beam at long propagation lengths may result in a reduction of the pump rate and an unsaturated XRL (see Methods). A similar low-gain behavior is also observed for the XRLs from the ^3^*P*_1_ → ^1^*S*_0_ transition in Kr^34+^ due to fast decay, and for the XRLs from the ^3^*S*_1_ → ^1^*S*_0_ transition in Xe^52+^ due to large broadening. These two XRLs are thus excluded from Table 4.

### Brightness and spatial coherence

The brightness is defined as the number of photons emitted per second within the 0.1% relative bandwidth under an emittance of 1 mm mrad and is calculated through^61^

1 $$B[{\rm{brt}}]=\frac{{10}^{17}}{1.6}\left(\frac{{I}_{{\rm{c}}}}{\hslash \varDelta \omega }\right)\left(\frac{{S}_{{\rm{focal}}}}{{\lambda }^{2}}\right),$$

where the units for the intensity *I*_c_, the bandwidth $\hslash \Delta \omega$, the focal size *S*_focal_ on the right hand side of Eq. (1) are the same as shown in Tables 3 and 4, with *λ* being the wavelength in units of nm. Since the Fresnel number is ${S}_{{\rm{focal}}}/{L}_{{\rm{c}}}\lambda \lesssim 1$ for our system, the outgoing XRL is transversely coherent, thus it can be described as a Gaussian beam, with *λ*^2^/*S*_focal_, in units of mrad^2^, being the solid angle the XRL emits into.

For XRLs from light elements, the calculated brightnesses, 3.2 × 10^27^ brt for Ne^8+ 1^*P*_1_, 8.1 × 10^30^ brt for Ar^16+ 3^*P*_1_ and 4.0 × 10^30^ brt for Ar^16+ 1^*P*_1_, are smaller than those in XFELs at the same photon energies. For XRLs from heavier elements such as Kr and Xe, however, with *B* = 6.1 × 10^33^ and 5.0 × 10^35^ brt, respectively, the brightnesses are significantly increased and can be larger than those available at XFELs, representing a significant advantage of our scheme in generating high-intensity fully coherent XRLs in the hard-x-ray regime.

The XRL photon fluxes can be calculated from Tables 3 and 4 as ${J}_{c}={I}_{c}/\hslash {\omega }_{0}$. In units of ph./s/cm^2^, this gives *J*_*c*_ = 3.0 × 10^28^, 1.0 × 10^30^ and 3.0 × 10^31^ for the three transitions in Table 3, respectively, indicating a relatively small conversion efficiency of x-ray photons from the XFEL pulses. However, with *J*_*c*_ = 2.1 × 10^31^ and 9.7 × 10^32^ for the two transitions in Table 4, respectively, a significantly larger efficiency is achieved for harder x-ray lasers.

## Discussion

The plasma used in our lasing scheme can be generated by illuminating a target with an intense optical laser^49,50^: first, the outer-shell electrons of the atoms in the gas undergo tunneling ionization in the presence of the laser; afterwards, their acceleration in the strong laser field and subsequent recollisions with the ions lead to the further ejection of more tightly bound electrons and the formation of dense highly charged ions^52–54^. The critical density and skin effect during the generation of dense plasmas can be overcome by either using optical lasers with shorter wavelengths or by adoption of plasma guiding schemes (see Supplemental Sec. C for additional details).

While an accurate prediction of the laser conditions for the production of the plasmas in Fig. 2 would require simulations involving many nonlinear light–matter interactions going beyond the scope of this article, here we provide a conservative estimation of the laser intensity, pulse duration and total power needed for plasma generation based on the FLYCHK atomic-physics plasma model and verified scaling laws^54^. FLYCHK is first used to obtain the electron temperature *T*_e_ required to maximize the density of Li-like ions in the plasma. For such values of *T*_e_, the required laser intensity at a given optical wavelength is estimated through the pondermotive scaling law^62,63^

2 $${T}_{{\rm{e}}}/{\rm{keV}}\approx 3.6\times {I}_{16}{\lambda }_{\mu }^{2},$$

where *I*_16_ is the laser intensity normalized to 1 × 10^16^ W cm^−2^, and *λ*_*μ*_ is the laser wavelength in units of *μ*m.

Nanoplasmas consisting of Ar^16+^ have been obtained from Ar clusters interacting with optical lasers^64^. Incoherent K_α_ emission from plasmas of He-like Ar ions, with a size varying from 20 to 35 nm, has been recorded for laser durations and intensities from 30 fs to 3.5 ps and 10^17^ W cm^−2^ to 10^14^ W cm^−2^, respectively^64^. To achieve the plasma conditions discussed in Fig. 2, a 2-picosecond-long, 0.44-TW, 800-nm optical laser with an intensity of 1.1 × 10^15^ W cm^−2^ and a total energy of 0.88 J per pulse is required (see Methods). High-repetition rate (up to 500 Hz), high-power (0.3 TW) picosecond lasers, with a single-pulse energy up to 1.5 J, have been used for the generation of table-top soft-XRLs^65,66^. Combining such laser systems with XFELs can produce high-repetition rate, high-intensity, narrow-band hard-XRLs with extended applications. The generation of plasmas consisting of highly charged Ne ions would be even less demanding, and could be achieved as well.

Though highly charged Kr^27+^ and Xe^48+^ have been produced by intense optical lasers^53^, the production of dense Kr^34+^ and Xe^52+^ plasmas has not been demonstrated yet. Following the same calculation procedure used for argon plasmas, we estimate that an optical laser with an intensity of 1.1 × 10^17^ W cm^−2^ and pulse duration of about 7 ps might be needed to achieve the plasma conditions for Xe^52+^ shown in Fig. 2. The estimated power and energy of 88 TW and 616 J, respectively, are currently not available at XFEL high-energy-density (HED) endstations. However, they can be reached at current high-power laser facilities^60^, where intense optical laser pulses with durations from 100 fs to 10 ps and energies of up to 1 kJ are typically available, indicating the feasibility to generate dense Xe^52+^ ions for x-ray lasing by using petawatt lasers in combination with XFEL facilities. Due to high thermal load^67^, the repetition rate of such petawatt lasers is typically below 10 Hz (ref. ^60^). However, a much higher repetition rate can be achieved when a large-scale fibre amplifier becomes available^68^.

Moreover, instead of using high-power optical lasers, a plasma consisting of highly charged ions can also be produced by an intense XFEL pulse^30,69^. The production of dense H-like ions directly by an XFEL pulse has already been demonstrated in Al plasmas^70,71^. However, the volumes of these plasmas are too small (around 9 *μ*m^2^ × 1 *μ*m) to obtain x-ray lasing. Due to large absorption, it is currently inefficient to use an XFEL pulse to generate the millimeter-long dense HCI plasmas required for high-intensity XRLs. For instance, in the XRL experiment with Cu foils, a significant attenuation of XFEL photons was observed after a propagation of 20 *μ*m (ref. ^23^). In contrast, the use of intense optical lasers proposed here can enable the generation of dense plasmas of HCIs with lengths up to tens of millimeter or even centimeters^24,25^, rendering them good candidates for demonstrating hard-XRLs with saturated intensities. Furthermore, though generating dense HCIs with XFEL pulses is possible for light elements, it is challenging to control the charge-state distribution and the pump rate separately. Using a laser-produced plasma instead of generating HCIs by the XFEL pump pulse itself^30^ results in a more efficient scheme, with more degrees of freedom to control and optimize the output of the XRL.

### Feasibility and limitations

We would like to point out that the plasma conditions presented in Fig. 2, such as temperature, density, and charge-state distributions, were obtained via steady-state atomic-physics plasma simulations, and represent a first approximation of the highly active medium^72,73^. Though there have been varieties of soft-x-ray lasers demonstrated from dense Ne- or Ni-like HCI plasmas^26–28^, direct experimental evidence of the plasma medium required by our lasing scheme, especially in the high-*Z* regime^53^, is still missing. Theoretically, an accurate prediction of the laser conditions needed to produce such plasmas would be beneficial, but this requires the inclusion of many complex and nonlinear light–matter interactions, which goes beyond the scope of the present article. Instead, our estimations provide a first insight into the possibility of producing the required plasmas with currently available or planned intense laser facilities. These promising results motivate future experimental investigations of high-energy-density physics^74^ at high-power laser and XFEL facilities worldwide^60,75–80^, as well as further theoretical studies of laser–matter interactions incorporating particle-in-cell (PIC) simulations^81,82^ and XRL modeling beyond the two-level approximation^42^.

### Summary and outlook

Our numerical simulations employing the Maxwell–Bloch equations, involving realistic parameters calculated from atomic structure theory and plasma simulations, show that the lasing scheme we put forward can be realized with currently available or planned high-power laser facilities at several XFEL endstations^75–80^. Such x-ray sources will enable novel studies of coherent light–matter interactions in atomic, molecular and solid-state systems^83^, e.g., x-ray quantum optics, high-resolution spectroscopy, and nonlinear x-ray scattering processes. As an example, there have been many experiments investigating the nonlinear interactions between x-rays and matter using intense XFEL pulses^8,38–44^, where several states are excited and many processes are involved simultaneously. However, the XRL proposed here can be used to excite an individual state, due to the good temporal coherence and saturated intensity, and could thus complement the capabilities at broadband, incoherent XFEL sources. This includes, e.g., x-ray stimulated Raman adiabatic passage for the coherent preparation of HCIs towards generating an x-ray frequency comb^9^, and resonant excitation of highly charged ions in an electron-beam ion trap for precision tests of quantum electrodynamics^84,85^.

Since the XRLs proposed here are seeded by spontaneous emissions from all the magnetic sublevels, similar to other XRLs, the photons are not polarized. However, one can gain control on the polarization by using a polarimeter^86^, or by seeding the XRL with an external polarized x-ray field^87,88^. Furthermore, a three/four-wave mixing scheme that couples the XRL with another optical/EUV laser can be investigated to tune the discrete XRL frequency in a wide range^89,90^. The XRLs put forward here can also be used to perform coherent x-ray pump-probe experiments for investigations of structural relaxation processes^91^.

## Methods

### Maxwell–Bloch theory

Maxwell–Bloch theory has been successfully applied to describe the amplification and absorption of light in active media^57^, including the development of soft x-ray lasers in laser-produced plasmas^92^. After the experimental realization of XRL pumped by an XFEL pulse, in ref. ^59^, Weninger and Rohringer successfully applied this theory to model the evolution of the corresponding XRL. In the following, we extend this theory to also include lasing from multipole transitions, i.e., *M*1 and *M*2 transitions. Assuming XFEL pulses propagating along the $\hat{x}$ direction, the evolution of the XRL field in the slowly varying envelope approximation is given by^57,59,92^

3 $$\frac{\partial {\mathscr{A}}(x,t)}{\partial t}+c\frac{\partial {\mathscr{A}}(x,t)}{\partial x}=i\frac{{\mu }_{0}\omega {c}^{2}}{2} {\mathcal F} (x,t),$$

where ${\mathscr{A}}(x,t)$ is either the envelope of the electric field ${\mathcal E} (x,t)$ or the envelope of the magnetic field ${\mathcal B} (x,t)$, depending on the specific transition. *μ*_0_ is the vacuum permeability and *ω* is the carrier frequency of the XRL. ${\mathcal F} (x,t)$ corresponds to the polarization field induced by ${\mathcal E} (x,t)$ for *E*1 transitions, or the magnetization field and magnetic-quadrupole field induced by ${\mathcal B} (x,t)$ for *M*1 transitions and *M*2 transitions, respectively.

Under the two-level approximation in He-like ions, we assume all Li-like ions are pumped into the corresponding upper lasing state of such transition by the XFEL pulse. Using $|{\rm{e}}\rangle$ and $|{\rm{g}}\rangle$ to represent the upper lasing state and the lower lasing state, respectively, the dynamics of the He-like ions are described by the Bloch equations of the density matrix

4 $$\begin{array}{rcl}{\dot{\rho }}_{{\rm{ee}}}(x,t) & = & -{\rm{Im}}[{\varOmega }^{\ast }(x,t){\rho }_{{\rm{eg}}}(x,t)]+{\sigma }_{0}{j}_{{\rm{xfel}}}(x,t){\rho }_{00}(x,t)\\  &  & -{\sigma }_{{\rm{e}}}{j}_{{\rm{xfel}}}(x,t){\rho }_{{\rm{ee}}}(x,t)-\Gamma {\rho }_{{\rm{ee}}}(x,t),\end{array}$$

5 $$\begin{array}{rcl}{\dot{\rho }}_{{\rm{eg}}}(x,t) & = & \frac{i}{2}{\varOmega }^{\ast }(x,t)({\rho }_{{\rm{ee}}}(x,t)-{\rho }_{{\rm{gg}}}(x,t))\\  &  & -\frac{\gamma }{2}{\rho }_{{\rm{eg}}}(x,t)+G(x,t),\end{array}$$

6 $$\begin{array}{rcl}{\dot{\rho }}_{{\rm{gg}}}(x,t) & = & {\rm{Im}}[{\varOmega }^{\ast }(x,t){\rho }_{{\rm{eg}}}(x,t)]-{\sigma }_{{\rm{g}}}{j}_{{\rm{xfel}}}(x,t){\rho }_{{\rm{gg}}}(x,t)\\  &  & +\Gamma {\rho }_{{\rm{ee}}}(x,t\mathrm{)}.\end{array}$$

The carrier frequency *ω* is chosen to be resonant with the lasing transition *ω*_0_. *ρ*_ee_ and *ρ*_gg_ are the populations of $|{\rm{e}}\rangle$ and $|{\rm{g}}\rangle$, and the off-diagonal term *ρ*_eg_ represents the coherence between the two lasing states. $\varOmega (x,t)=\wp  {\mathcal E} (x,t)/\hslash$ for an *E*1 transition (or $\varOmega (x,t)=m {\mathcal B} (x,t)/\hslash$ for an *M*1 transition, and $\varOmega (x,t)={k}_{0}{q}_{yx} {\mathcal B} (x,t)/\hslash$ for an *M*2 transition^93^) is the time- and space-dependent Rabi frequency, with $\wp$ the electric-dipole moment, *m* the magnetic-dipole moment, *k*_0_ the wavenumber and *q*_*yx*_ the *yx*-component of the magnetic-quadrupole tensor. For a magnetic-quadrupole transition, *k*_0_*q*_*yx*_ represents the effective magnetic-dipole moment coupling with the magnetic field. XFEL pumping of the $|{\rm{e}}\rangle$ state from Li-like ions is accounted for through the second term in the right-hand side of Eq. (4), with *ρ*_00_ being the population of Li-like ions, *j*_xfel_ the photon flux of the XFEL pump pulse, and *σ*_0_ the K-shell photoionization cross section of the pump process (1*s*^2^2*l* → 1*s*2*l*). The XFEL pulse also depletes the $|{\rm{e}}\rangle$ and $|{\rm{g}}\rangle$ states, as modeled by the third term on the right-hand side of Eq. (4) and the second term on the right-hand side of Eq. (6), respectively, with *σ*_e_ and *σ*_g_ being the corresponding photoionization cross sections. In Eq. (4) and (6), Γ*ρ*_ee_(*x*, *t*) describes spontaneous emission at rate Γ. In Eq. (5), the parameter

7 $$\gamma =\Gamma +\Delta {\omega }_{{\rm{e}}-{\rm{i}}}+({\sigma }_{{\rm{e}}}+{\sigma }_{{\rm{g}}}){j}_{{\rm{xfel}}}(x,t)$$

models the three contributions to the decay of the off-diagonal elements: Γ is the decoherence originating from spontaneous photon emission; the second term Δ*ω*_e−i_ accounts for the broadening from electron–ion collisions^56^; and the final term describes the contribution from depletion of the total population of He-like ions. To model seeding from spontaneous emission, *G*(*x*, *t*) in Eq. (5) is a Gaussian white-noise term added phenomenologically, which satisfies $\langle {G}^{\ast }(x,t)G(x,t{\prime} )\rangle =K(x,t)\delta (t-t{\prime} )$. For *E*1 transitions, one has^92^

8 $$K(x,t)=\frac{{\varepsilon }_{0}\hslash {\omega }_{0}}{{N}_{{\rm{ion}}}{\wp }^{2}}\frac{\Theta }{8\pi }\frac{{\gamma }^{2}}{{\omega }_{0}^{2}}\Gamma {\rho }_{{\rm{ee}}}(x,t),$$

where $\Theta  \sim {S}_{{\rm{focal}}}/{L}^{2}$ represents the solid angle in the forward direction, with *S*_focal_ the focal size of the XFEL pulse and *L* the length of the plasma.

The coupling between the Maxwell equations and the Bloch equations is given through the induced fields ${\mathcal F} =-2{N}_{{\rm{ion}}}d{\rho }_{{\rm{eg}}}$, with $d=\wp ,\,m,\,{k}_{0}{q}_{yx}$ being the effective dipole and quadrupole moments for *E*1, *M*1, and *M*2 transitions, respectively^57^. Absorption of the XFEL pulse by the ions is included through the rate equations

9 $$\frac{\partial {j}_{{\rm{xfel}}}}{\partial z}=-\sum _{{\rm{k}}}\,{\sigma }_{{\rm{k}}}{\rho }_{{\rm{kk}}}{N}_{{\rm{ion}}}\,{j}_{{\rm{xfel}}},$$

where k represents any state that can be ionized by the XFEL pulse. The effect of other charge states, namely the C-, B- and Be-like ions, is also accounted for via rate equations.

### Steady-state FLYCHK plasma simulation

The generation of HCI plasmas consists of two distinct stages^52–54^: at the initial stage, it is dominated by nonlinear processes such as field-induced ionization and tunneling ionization leading to the production of lower charge states by ejection of the outermost electrons; this usually takes a few femtoseconds or less; after that, the ionization potential is too deep to be reached and these nonlinear processes become inefficient. Therefore, in the second stage, linear processes such as collisional ionization induced by hot electrons become dominant in the production of higher charged ions. Due to the small size of the plasma, photons emitted by spontaneous emission, radiative recombination and bremsstrahlung immediately escape the plasma, leading to a radiative loss and a decrease of the electron temperature. The role of the intense laser at this stage is to stabilize the electron temperature via reaccelerating the electrons to compensate for the radiative loss.

At a later stage, the plasma will evolve into a steady-state where linear processes such as collisional ionization, excitation, radiative recombination and spontaneous emission equilibrate with each other, and collisional-radiative atomic plasma codes such as FLYCHK can be used to simulate the corresponding plasma conditions under given electron temperature and density. Although the FLYCHK code does not include the optical laser field, the effects of the field on the HCI plasma are effectively accounted for in our FLYCHK simulation via the assumption of a stable thermal electron temperature approximated by the pondermotive scaling law in Eq. (2). Such a Maxwellian electron distribution has found to be a good approximation in previous simulations of laser-produced plasmas^62,63^ and is used here to show the feasibility of our scheme. Nevertheless, the optical field will modify the electron distributions, and PIC simulations^81,82^ might be needed to estimate the electron distributions for more accurate FLYCHK simulations.

The background radiation field in the FLYCHK simulations is neglected due to the small lateral size of the elongated plasma. This is mainly because, for a linear plasma with a ~10 *μ*m^2^ cross-sectional area, photons emitted by the ions and electrons immediately escape from the plasma with negligible reabsorption. Therefore, the radiative temperature is set to be zero for all the FLYCHK simulations, i.e., no blackbody radiation field is considered. The validity of such a treatment can be confirmed from the FLYCHK simulations, where the calculated optical depth is ~10^−4^ or smaller for photon energies beyond 10 eV.

### Line broadening effects in the plasma

Doppler broadenings shown in Tables 3 and 4 are calculated as

10 $$\Delta {\omega }_{{\rm{D}}}=\sqrt{\frac{8\,\mathrm{ln}\,2{k}_{{\rm{B}}}{T}_{{\rm{i}}}}{{m}_{{\rm{i}}}{c}^{2}}}{\omega }_{0},$$

with *k*_B_ being the Boltzmann constant, *T*_i_ the ion temperature and *m*_i_ the mass of the He-like ions. This is not significant for light ions, but it becomes dominant for heavy ions like Kr^34+^ and Xe^52+^. For the plasma conditions used in the main text, the electron–ion equilibration time is in the range of hundreds of picoseconds^94^. Therefore, the ion temperature is assumed to be not significantly modified during the plasma generation and the lasing process, which is around a few picoseconds or less.

The electron-impact broadening is given by^56^

11 $$\Delta {\omega }_{{\rm{e}}-{\rm{i}}}=-\frac{16}{3{\bar{v}}_{{\rm{e}}}}\frac{{N}_{{\rm{e}}}{\hslash }^{2}}{{Z}_{{\rm{i}}}^{2}{m}_{{\rm{e}}}^{2}}\,\mathrm{ln}\,\Lambda \,\langle {\boldsymbol{rr}}\rangle ,$$

with ${\bar{v}}_{{\rm{e}}}=\sqrt{8{k}_{{\rm{B}}}{T}_{{\rm{e}}}/\pi {m}_{{\rm{e}}}}$ being the average thermal velocity of the electrons in the plasma and *N*_e_ the electron density, and *Z*_i_ the charge number of the ions. *T*_e_ and *m*_e_ are the electron temperature and electron mass, respectively. ln Λ ~ 10 is the Coulomb logarithm and **rr** is a tensor with **r** being the dipole operator of the bound electrons in the ions. Such broadening is significant only for light ions such as Ne^8+^ and Ar^16+^ with lower electron temperatures, but becomes negligible compared to Δ*ω*_D_ for heavy ions and higher electron temperatures.

The quadratic Stark broadening from ion–ion interaction is calculated through^56^

12 $$\Delta {\omega }_{{\rm{i}}-{\rm{i}}}=\alpha \overline{{F}^{2}},$$

with

13 $$\alpha =-\,\frac{1}{4\pi \hslash }\sum _{k\ne j}\,{\wp }_{jk}^{2}/(e{E}_{kj}^{2}),$$

14 $$\overline{{F}^{2}}=4{Z}_{{\rm{p}}}^{2}{e}^{2}{N}_{{\rm{ion}}}^{\frac{4}{3}}/{(\pi {\varepsilon }_{0})}^{2},$$

where *α* is the quadratic Stark coefficient which is different for each transition, and $\overline{{F}^{2}}$ is the mean-square electric-field strength generated by nearby perturbing ions with charge number *Z*_p_. ${\wp }_{jk}$ and *E*_*jk*_ are the electric-dipole moment and energy difference between the states $|{\rm{j}}\rangle$ and $|{\rm{k}}\rangle$, respectively. Populations in different charge states *Z*_p_, as predicted from FLYCHK simulations, are also fully taken into account by using $\langle {Z}_{{\rm{p}}}^{2}\rangle$ in the prediction of the mean-square field $\overline{{F}^{2}}$. Δ*ω*_i−i_, in general, is negligible for *N*_ion_ < 10^19^ cm^−3^, but becomes large for dense ion gases.

In general, the natural broadening and electron–ion impact broadening are homogeneous for each ion, yielding a Lorentzian line profile. The Doppler broadening and ion–ion Stark broadening, on the other hand, are inhomogeneous for different ions, and result in a Gaussian profile. For systems involving both homogeneous and inhomogeneous broadenings, as it is the case here, the real spectrum has a Voigt line shape given by the convolution of the Lorentzian and Gaussian profiles, with a FWHM Δ*ω*_V_ approximately given by^95^

15 $$\Delta {\omega }_{{\rm{V}}}=0.5364\Delta {\omega }_{{\rm{L}}}+\sqrt{0.2166\Delta {\omega }_{{\rm{L}}}^{2}+\Delta {\omega }_{{\rm{G}}}^{2}}\mathrm{}.$$

Here, $\Delta {\omega }_{{\rm{L}}}=\varGamma +\Delta {\omega }_{{\rm{e}}-{\rm{i}}}$ is the FWHM of the Lorentzian function, and $\Delta {\omega }_{{\rm{G}}}=\Delta {\omega }_{{\rm{D}}}+\Delta {\omega }_{{\rm{i}}-{\rm{i}}}$ is the FWHM of the Gaussian function.

Numerical simulations of the lasing process accounting for the inhomogeneous broadening should take into account the distributions of the thermal velocity as well as Stark shift of the ions. This renders the simulations time consuming. However, Eq. (15) shows that

16 $$\begin{array}{lll}\Delta {\omega }_{{\rm{V}}} &  \sim  & \Delta {\omega }_{{\rm{L}}}+\Delta {\omega }_{{\rm{G}}}\\  & = & \Gamma +\Delta {\omega }_{{\rm{e}}-{\rm{i}}}+\Delta {\omega }_{{\rm{D}}}+\Delta {\omega }_{{\rm{i}}-{\rm{i}}},\end{array}$$

so that, for the sake of simplicity, we can approximate the parameter in Eq. (7) as

17 $$\begin{array}{rcl}\gamma  & = & \Gamma +\Delta {\omega }_{{\rm{e}}-{\rm{i}}}+\Delta {\omega }_{{\rm{D}}}+\Delta {\omega }_{{\rm{i}}-{\rm{i}}}\\  &  & +({\sigma }_{{\rm{e}}}+{\sigma }_{{\rm{g}}}){j}_{{\rm{xfel}}}(x,t).\end{array}$$

With this approximation, the distribution of the ions over different thermal velocities and Stark shifts is included in the Maxwell–Bloch equations. This simplification may lead to a maximum of 25% overestimate of the bandwidth compared to the Voigt bandwidth Δ*ω*_V_ in the simulations. However, this will not change our conclusions in the main text.

Furthermore, the electron density and charge-state distribution are changing during the pumping and lasing processes, leading to a time-dependent broadening effect. This effect, accounting for less than 10% of the total broadening, is neglected in our simulation.

### Numerical XRL simulations

We simulate the lasing process by solving the Maxwell–Bloch equations numerically in retarded-time coordinates, with a time step of *δt* = 0.001*τ* and a grid size of *δx* = *cδt*, for 1,000 different realizations of SASE XFEL pulses. Small time-dependent changes in the broadenings due to modified charge-state distributions, densities and temperatures are neglected. Compared to Γ and Δ*ω*_e−i_ which are homogeneous for each ion, Δ*ω*_D_ and Δ*ω*_i−i_ are inhomogeneous broadenings. Without modifying the main conclusions, they are effectively accounted for in the simulation by redefining the decoherence rate in Eq. (7) according to Eq. (17). Values of the initial populations of the states in different ions under given plasma conditions are taken from the charge-state distribution shown in Fig. 2, with negligible populations in the excited states^51^.

The XFEL bandwidths listed in Table 3 are chosen based on realistic parameters at XFEL facilities in operation or under construction. However, for the case of Kr^34+^ and Xe^52+^ in Table 4, the time step employed in our numerical calculations of the Maxwell–Bloch equations is *δt* = 0.95 fs (*δt* = 0.0001*τ*) for Kr^34+^ and *δt* = 0.34 fs for Xe^52+^, respectively. This time step *δt* sets the limit on the smallest coherence time, thus the largest bandwidth, we can achieve in modeling the SASE XFEL pulse. Nevertheless, since the photoionization cross sections do not change significantly within the 0.1% relative bandwidth of the XFEL peak^47^, given the same flux, the simulations with larger bandwidths would provide the same results as we obtained in Table 4. Therefore, when we compare the bandwidth of the XRLs and XFEL pulses, we refer to the realistic bandwidth measured at XFEL facilities and not to the values used in Table 4.

### XFEL focal size

We assume that the XFEL pulse can be treated as a Gaussian beam whose transverse intensity is given by a Gaussian function. To ensure that the beam divergence does not reduce the intensity significantly after a propagation distance of *L*_c_ shown in Tables 3 and 4, the focal depth of the XFEL beam should be comparable with the optimal plasma length *L*_c_. Therefore, the minimum size of the XFEL focal spot for each transition shall be determined according to

18 $${S}_{{\rm{focal}}}=\frac{{L}_{{\rm{c}}}}{2}{\lambda }_{{\rm{xfel}}}\mathrm{}.$$

The calculated values, in units of *μm*^2^, are 1.4, 0.5, 0.05, 10.5 and 0.13 for the systems listed in Tables 3 and 4, respectively.

Another factor determining the focal size is the photon number. The maximum values of photons per pulse at given XFEL photon energies can be estimated from Fig. 19 in ref. ^4^, which are around 2 × 10^14^, 5 × 10^13^, 4 × 10^12^ and 1 × 10^12^ for XFEL photon energies of 1.2, 4.1, 17 and 40 keV, respectively. Given the photon flux and pulse duration listed in Tables 3 and 4, one obtains another bound for the XFEL focal size via

19 $${S}_{{\rm{focal}}}=\frac{{\rm{photons}}\,{\rm{per}}\,{\rm{pulse}}}{({\rm{photon}}\,{\rm{flux}})\cdot ({\rm{pulse}}\,{\rm{duration}})},$$

which gives the values of 85, 1.8, 12, 0.1 and 0.02 *μm*^2^ for the corresponding systems, respectively. Comparing the two bounds, we choose to use the focal sizes of 1.5, 1.0, 0.1, 0.1 and 0.13 *μm*^2^, respectively, as listed in Tables 3 and 4.

### Laser parameters required for the generation of Ar plasma

In order to show how the plasmas discussed in this paper can be produced with current laser facilities, we provide a conservative estimation of the laser conditions necessary to generate, e.g., a plasma consisting of highly charged Ar ions, for lasing from the ^3^*P*_1_ → ^1^*S*_0_ transition in Ar^16+^. This requires one to consider the time and energy needed to produce the plasma from a neutral gas and, at the same time, the time and total energy required for the plasma to evolve into a steady-state characterized by the corresponding charge-state distribution (red line) shown in Fig. 2.

First, the energy per electron necessary to generate such plasma can be conservatively estimated as the sum of the kinetic energy of the electron gas (*T*_e_ = 250 eV) and the average ionization energy *W*_0_ = 366 eV per electron. The latter is determined by the mean value of first, second, …, sixteenth ionization energies, and calculated based on the ionization energy data from the NIST Atomic Spectra Database^96^. Thus, the total energy deposited in the plasma can be approximated as

20 $$W\approx ({T}_{{\rm{e}}}+{W}_{0}){N}_{{\rm{e}}}SL\mathrm{}.$$

*N*_*e*_ is the electron density, while *S* and *L* are the cross-sectional area and the length of the plasma, respectively. For simplicity, we assume that all the ions in the plasma are He-like ions such that *N*_e_ ≈ 16 *N*_ion_. Supposing that an intense optical laser with a line focus of 10 *μ*m × 4 mm (given the optimal length of 3.3 mm shown in Table 3) illuminates the Ar gas of a thickness of 1 *μm*, we estimate *W* ≈ 1.6 mJ. With an absorption rate of the laser pulse of 10% (ref. ^97^), this requires a laser of an energy of 16 mJ. Based on Eq. (2), obtaining such an Ar plasma from a laser of a wavelength of 800 nm requires a laser intensity of about 1.1 × 10^15^ W cm^−2^. With a 10 *μ*m × 4 mm linear geometry, the power needed for such an experiment is estimated as 0.44 TW. For an energy of 16 mJ, this implies a pulse duration of ~36 fs.

However, in order to let the plasma evolve into a steady state characterized by the corresponding charge-state distribution (red line in Fig. 2), the optical laser is required also to compensate for the energy loss from processes such as recombination and to prevent the electron gas from cooling down^73,98^. Therefore, the pulse duration of the optical laser should be long enough to cover the whole plasma generation, equilibration and lasing processes. For the plasma conditions shown in Fig. 2, the collisional-ionization times obtained from the FLYCHK simulations are of the order of a few picoseconds or fractions thereof. Together with the subsequent 1 ps lasing process shown in Fig. 4a, the whole process lasts approximately 2 ps for the corresponding Ar plasma. Given the 0.44 TW power calculated above, such pulse duration corresponds to a total energy of 0.88 J, which is achievable at currently existing high-power laser facilities^60,65,66^.

## Supplementary information

Supplementary Information.

## Data Availability

The data that support the findings of this study are available from the corresponding author upon reasonable request.

## Code availability

The codes used to generate the results reported in this study are available from the corresponding author upon reasonable request.
