# Supplementary material for: Narrow-band hard-x-ray lasing with highly charged ions
Source: Sci Rep. 2020 Jun 10;10:9439. doi: 10.1038/s41598-020-65477-0 (PMC7287111; doi:10.1038/s41598-020-65477-0)
Supplement: Supplementary file 1 — Supplementary Information. [file 41598_2020_65477_MOESM1_ESM.pdf]

Narrow-band hard-x-ray lasing with highly charged ions: Supplemental Material

Chunhai Lyu, Stefano M. Cavaletto,* Christoph H. Keitel, and Zoltán Harman
Max-Planck-Institut für Kernphysik, Saupfercheckweg 1, 69117 Heidelberg, Germany

(Dated: April 5, 2020)

A. PHOTOIONIZATION CHANNELS IN LI-LIKE AND HE-LIKE IONS

In addition to the channel that directly pumps the upper lasing state shown in the second column of Table 2 in the main text, there are also other channels. In Table S1 we summarize the main results.

Table S1. **Photoionization channels in Li-like ions.** The energy thresholds and cross sections σ are calculated from the LANL Atomic Physics Codes [1], with the XFEL photon energy tuned above the $1s^2 \to 1s$ ionization channel. The channel denoted by a $\times$ is forbidden by single-photon ionization.

channel		Ne		Ar		Kr		Xe	
initial state	final state	threshold (keV)	σ (kb)	threshold (keV)	σ (kb)	threshold (keV)	σ (kb)	threshold (keV)	σ (kb)
Li-like	He-like								
$1s^2 2p\ ^2P_{1/2}$	$1s2p\ ^3P_0$	1.138	37.2	4.012	11.4	17.076	2.5	39.922	1.1
	$1s2p\ ^3P_1$	1.138	76.6	4.013	24.6	17.084	7.3	39.936	3.2
	$1s2p\ ^3P_2$	1.139	$\times$	4.016	$\times$	17.149	$\times$	40.334	$\times$
	$1s2p\ ^1P_1$	1.145	35.8	4.029	7.3	17.172	0.2	40.365	0
	total		149.6		43.3		10.0		4.3
$1s^2 2p\ ^2P_{3/2}$	$1s2p\ ^3P_0$	1.138	$\times$	4.009	$\times$	17.011	$\times$	39.573	$\times$
	$1s2p\ ^3P_1$	1.138	17.5	4.010	3.6	17.018	0.0	39.551	0.0
	$1s2p\ ^3P_2$	1.138	93.0	4.013	27.4	17.084	6.2	39.949	2.7
	$1s2p\ ^1P_1$	1.145	39.1	4.025	12.4	17.106	3.7	39.981	1.6
	total		149.6		43.4		9.8		4.3
He-like	H-like								
$1s^2\ ^1S_0$	$1s\ ^1S_{1/2}$	1.196	151.2	4.123	44.6	17.315	10.9	40.302	5.3

B. TRAVELING-WAVE SETUP

Since an XFEL pulse requires a time of ~ 13 ps to cross a medium of a length of $L \approx 4\,\mathrm{mm}$, plasma expansion may still take place on a comparable time scale of $\tau_\mathrm{p} \sim 10$ ps. For such a case, a traveling-wave setup, as depicted in Fig. S1, could be used [2–4]. Thereby, the plasma along the amplification path is generated shortly before the arrival of the XFEL pulse. Such setup would allow one to avoid an excessive expansion of the plasma before it is pumped by the XFEL pulse.

* Corresponding author: smcavaletto@gmail.com.; Present address: Department of Chemistry and Department of Physics and Astronomy, University of California, Irvine, CA 92697-2025, USA.

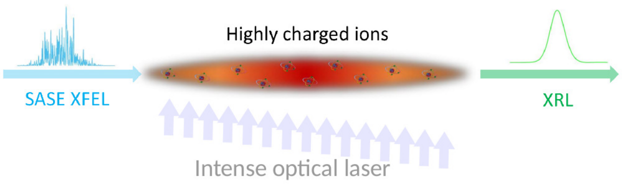

Figure S1. **Traveling-wave geometry.** The wavefront of the intense optical laser arrives at the target with a time delay.

C. PLASMA SKIN EFFECT

Most of the plasmas listed in Fig.2 in the main text are underdense, i.e., their electron density is below the critical density of $N_{\rm e,crit} = \varepsilon_0 m_{\rm e} \omega_{\rm op}^2/e^2 = 1.7 \times 10^{21}\,{\rm cm}^{-3}$ for an optical pulse with a wavelength of 800 nm [5]. Here, $\omega_{\rm op}$ is the angular frequency of the optical laser, and e, ε_0 and $m_{\rm e}$ are the elementary charge, vacuum permittivity and electron rest mass, respectively. As a result, in this so-called linear regime, the optical pulse will propagate through the medium without encountering further obstacles. For Ar and Xe, where $N_{\rm e} = 4.0 \times 10^{21}\,{\rm cm}^{-3}$ and $1.4 \times 10^{23}\,{\rm cm}^{-3}$ are larger than $N_{\rm e,crit}$, however, the propagation of the optical pulse in the gas will stop at lengths comparable to the plasma skin depth [5],

$$\xi = \frac{c}{\sqrt{\omega_{\rm pl}^2 - \omega_{\rm op}^2}}, \tag{S1}$$

with c the speed of light and $\omega_{\rm pl} = \sqrt{N_{\rm e} e^2/\varepsilon_0 m_{\rm e}}$ the plasma frequency. The definition in Eq. (S1), holding when $\omega_{\rm op} < \omega_{\rm pl}$, gives the penetration depth at which the amplitude of the optical laser was reduced by $1/e$ (here, $e = 2.71828...$ is the Euler's number, and should not be confused with the elementary charge indicated by the same symbol). Therefore, the skin depth ξ, which is 113 nm and 14 nm for Ar and Xe respectively, will determine the region where the x-ray lasing scheme is effective. This is also one of the reasons why the optical laser should irradiate the target laterally to create a millimeter-long plasma.

There are several ways that can be used to overcome the skin effect. One option is to use an optical laser with a shorter wavelength, e.g., 259 nm. With a critical density of $N'_{\rm e,crit} = 1.7 \times 10^{22}\,{\rm cm}^{-3}$, the Ar plasmas considered in this work would then be underdense and could be penetrated by a 259 nm optical laser. For a Xe plasma, it would result in an electron density of $8.2N'_{\rm e,crit}$ and a skin depth of 15 nm. Nevertheless, different guiding schemes [6] can be used to produce Xe plasmas with a larger cross-sectional area for the XRLs.

D. ADDITIONAL SIMULATION RESULTS FOR HOMOGENEOUS PLASMA CONDITIONS

Simulation results of the intensity profile and spectrum as a function of propagation length for shots 2–4 in Fig. 3 in the main text, as well as the intensity profile and spectrum averaged over 1,000 simulations, are displayed in Figs. S2–S4 and Fig. S5, respectively. Shot-to-shot differences are a consequence of the noisy spontaneous-emission seeding and the random SASE-XFEL-pulse profiles used, shown in Figs. 3e-f in the main text.

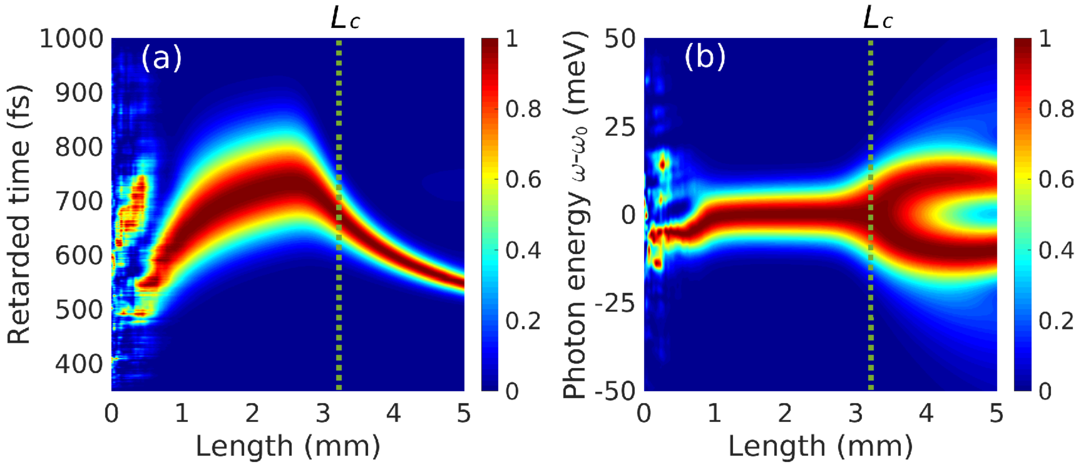

Figure S2. **Evolution of the normalized XRL intensity and spectrum (shot 2).** Results for SASE-pulse shot 2 in Fig. 3f in the main text; green dotted lines in Figs. 3c,d in the main text. **a**, Intensity shown as a function of retarded time and propagation length. **b**, Power spectrum displayed as a function of photon energy and propagation length. For a given length, the intensity and spectrum are normalized to the maximum value of the corresponding profiles at such length. The vertical dotted lines indicate the characteristic length L_c defined in the main text. The decrease in the XRL bandwidth at the end of the medium is a result of XFEL absorption. Figure reproduced from C. Lyu's Ph.D. thesis [7].

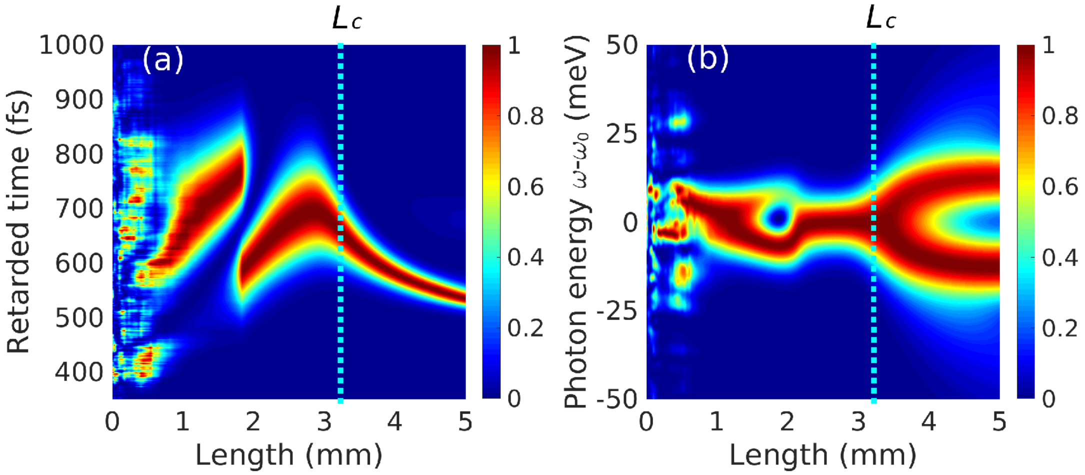

Figure S3. **Same as Fig. S2 for shot 3.** Results for SASE-pulse shot 3 in Fig. 3g in the main text; blue dotted lines in Figs. 3c,d in the main text. The exotic structures in both intensity and spectrum around $L = 2$ mm originate from XFEL photoionization of the upper lasing state. The decay of this state is different during and after the XFEL pulse. This renders it possible that two peaks develop and propagate before saturation. Figure reproduced from C. Lyu's Ph.D. thesis [7].

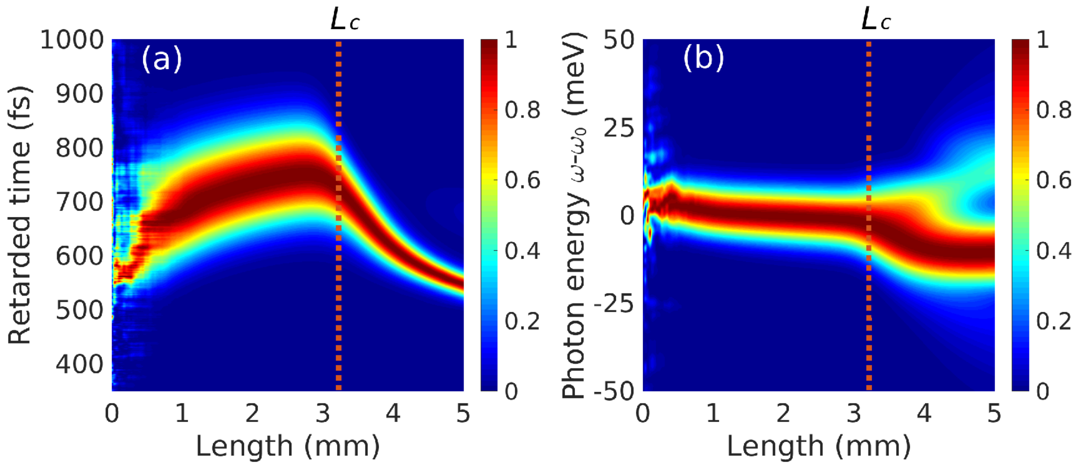

Figure S4. **Same as Fig. S2 for shot 4.** Results for SASE-pulse shot 4 in Fig. 3h in the main text; red dotted lines in Figs. 3c,d in the main text. A slight shift of the peak of the spectrum from ω_0 is here apparent. Figure reproduced from C. Lyu's Ph.D. thesis [7].

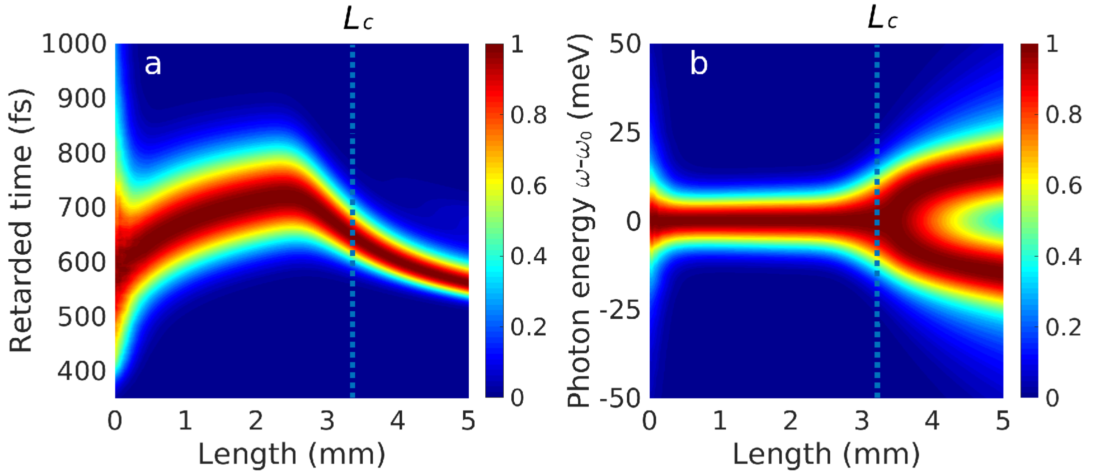

Figure S5. **Evolution of the normalized XRL intensity and spectrum averaged over 1,000 realizations of SASE XFEL pulses.** Intensity and spectra evaluated at the characteristic length L_c are exhibited by the solid lines in Figs. 3c,d in the main text. The chaotic features for small propagation lengths, which are shown for single simulations in Figs. 3a,b in the main text and in Figs. S2–S4 above, become smooth here. Figure reproduced from C. Lyu's Ph.D. thesis [7].

E. SIMULATION FOR INHOMOGENEOUS PLASMA CONDITIONS

In order to prove the effectiveness of our lasing scheme in realistic conditions, we perform simulations in the presence of an inhomogeneous plasma. This is achieved by modeling ion density and total broadening effects by the time- and space-dependent functions $f(t,x)\,N_{\rm i}$ and $f(t,x)\,\gamma$, respectively, with $N_{\rm i} = 2.5 \times 10^{19}\,{\rm cm}^{-3}$ and where γ is calculated from Eq. (16) in the main text. Figure S6 displays results on the evolution of the XRL from Ar^{16+} $^3P_1$, obtained by averaging over 1,000 different realizations of the XFEL pump pulse and of the time- and space-dependent factor $f(t,x)$, with one realization of $f(t,x)$ is exhibited in Fig. S6a. The inhomogeneity function $f(t,x)$, with $\langle f(t,x)\rangle = 1$, is assumed to be a Gaussian noise with a finite correlation time of 0.16τ at each x, where τ is the natural lifetime of the upper lasing state. The correlation along the plasma, i.e. along x, at given time t is assumed to be a δ-function for the convenience of numerical implementation.

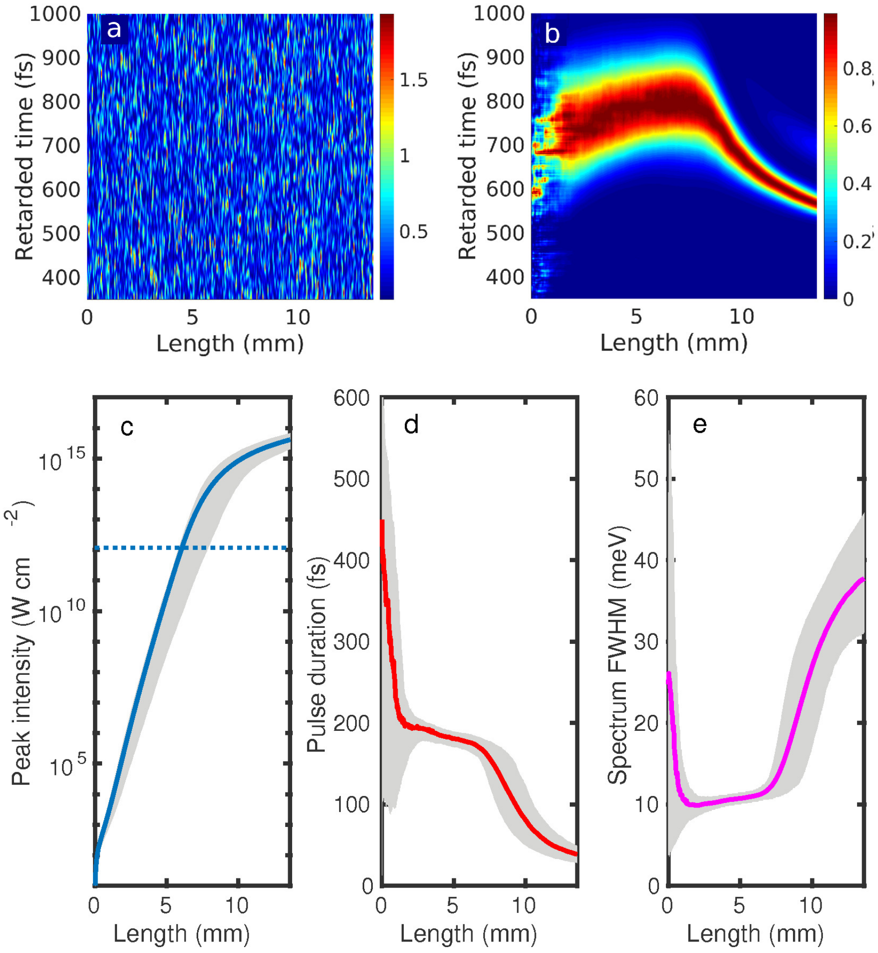

Figure S6. **Evolution of the XRLs over 1,000 simulations** (Ar^{16+} $^3P_1$) **for inhomogeneous plasma conditions**. **a**, Time- and space-dependent factor $f(t,x)$ used to simulate inhomogeneous plasma density and broadening effects in the medium. **b**, XRL intensity normalized to the maximum value of the corresponding profiles at that length. **c**-**e**, Peak intensity, pulse duration and spectral full width at half maximum (FWHM) of the XRL as a function of propagation length. Figure adapted from C. Lyu's Ph.D. thesis [7].

As shown in Fig. S6, the effectiveness of our X-ray lasing scheme is not compromised by the inhomogeneous conditions through the plasma. The saturation intensity is the same as in our previous simulations for a homogeneous plasma. However, our new simulations show that slightly longer media are necessary to reach these saturation intensities, as one can deduce by comparing Fig. S6 with our previously obtained results from Figs. 2 and 3 in the main text. This is due to the presence of regions with relatively smaller density, which results in a smaller gain coefficient and slower X-ray amplification. The lengths necessary to reach saturation are still of the same order of magnitude as in the case of constant plasma parameters. Faster amplification may still be achieved by increasing the average density in the medium.

In spite of averaging over results obtained for different inhomogeneous media (i.e., employing different realizations of the function $f(t,x)$), the pulse duration and spectrum are very well centered around their average values. Compared to previous simulations with constant plasma parameters, the gray areas (displaying the distribution areas of the results over 1,000 simulations) are larger only for propagation lengths smaller than L_1. For larger propagation lengths, the X-ray laser is transform-limited and its spectral width is smaller than the width γ including all broadening effects. This renders it less sensitive to the actual value of γ, and hence less sensitive to its variation in an inhomogeneous plasma. The narrow bandwidth of the X-ray laser is a consequence of gain narrowing, and its value is more closely associated with the gain coefficient in the medium than with the precise value of the width γ. The narrow-band condition is therefore feasible, even when inhomogeneous ions create space-dependent broadening effects.

F. OTHER XRL CHARACTERISTICS

Parameters like intensity, bandwidth and plasma lengths are shown in Tables 3 and 4 in the main text. Other parameters such as peak power, pulse duration Δt, energy per pulse, photon number per pulse and brightness are shown in Table S2.

Table S2. **Extended XRL characteristics.** The units of the brightness is brt = photons/s/mm^2/mrad^2/0.1%bandwidth.

Lasers $1s2l$	Peak power (MW)	Δt (fs)	Energy (μJ per pulse)	Photons (per pulse)	Photon flux ph./s/cm^2	Brightness (brt)
Ne^{8+} $^1P_1$	0.068	26	0.002	1.2×10^7	3.0×10^{28}	3.2×10^{27}
Ar^{16+} $^3P_1$	5.0	87	0.435	8.7×10^8	1.0×10^{30}	8.1×10^{30}
Ar^{16+} $^1P_1$	15	1.9	0.028	5.7×10^7	3.0×10^{31}	4.0×10^{30}
Kr^{34+} $^3P_2$	43	757	32.6	1.6×10^{10}	2.1×10^{31}	6.1×10^{33}
Xe^{52+} $^3P_2$	6110	23	140.5	2.9×10^{10}	9.7×10^{32}	5.0×10^{35}

The brightnesses are obtained based on the formula [8]:

$$\begin{aligned} B &= (\text{photon flux}) \left(\frac{0.1\%\ \omega}{\Delta\omega}\right) \left(\frac{1}{\text{solid angle}}\right) \\ &= 10^{-3} \left(\frac{\text{intensity}}{\hbar\Delta\omega}\right) \left(\frac{1}{\text{solid angle}}\right) \\ &= 10^{-3} \left(\frac{I_\mathrm{c}}{\hbar\Delta\omega}\right) \left(\frac{S_\mathrm{focal}}{\lambda^2}\right). \end{aligned} \tag{S2}$$

All the quantities in Eq. (S2) are expressed in the international system of units, with I_c being the intensity, $\hbar\Delta\omega$ the energy bandwidth, S_focal the focal area of the XFEL pulse and $\lambda = 2\pi c/\omega$ the wavelength of the XRL. Thus, $\lambda^2/S_\mathrm{focal}$ represents the solid angle into which the XRL is emitted at the end of the plasma, where a Gaussian transverse profile for the pulse is assumed. For completeness, we derive the formula with the units used in this paper as

$$B[\text{brt}] = \frac{10^{17}}{1.6} \left(\frac{I_\mathrm{c}[\text{W/cm}^2]}{\hbar\Delta\omega[\text{meV}]}\right) \left(\frac{S_\mathrm{focal}[\mu\text{m}^2]}{(\lambda[\text{nm}])^2}\right). \tag{S3}$$

Furthermore, the peak power can be calculated via the formula

$$\begin{aligned} \text{peak power} &= (\text{peak intensity}) \cdot (\text{focal area}) \\ &= 10^{-8} I_\mathrm{c}[\text{W/cm}^2] \cdot S_\mathrm{focal}[\mu\text{m}^2], \end{aligned} \tag{S4}$$

and the pulse energy can be estimated via

$$\text{pulse energy} = (\text{peak power}) \cdot (\text{pulse duration}), \tag{S5}$$

which results in a photon number per pulse as

$$\text{number of photons} = \frac{\text{pulse energy}}{\hbar\omega_0}, \tag{S6}$$

and photon flux as

$$\text{photon flux} = \frac{\text{number of photons}}{\text{pulse duration} \cdot (\text{focal area})} = \frac{\text{intensity}}{\text{photon energy}}. \tag{S7}$$

With these formulas, the values for the peak power, pulse energy, number of photons, photon flux and brightness are calculated and listed in Table S2.

[1] LANL Atomic Physics Codes, URL `http://aphysics2.lanl.gov/cgi-bin/ION/runlanl08f.pl`.

[2] H. Daido, Rep. Prog. Phys. **65**, 1513 (2002).
[3] T. Kawachi *et al.*, Phys. Rev. A **66**, 033815 (2002).
[4] D. Alessi *et al.*, Phys. Rev. X **1**, 021023 (2011).
[5] T. V. Liseykina and D. Bauer, Phys. Rev. Lett. **110**, 145003 (2013).
[6] N. Lemos, Sci. Rep. **8**, 3165 (2018) (and the references therein).
[7] Lyu C. *Narrow-band hard-X-ray lasing.* Ph.D. Thesis, Heidelberg University, Germany (2018), URL `http://archiv.ub.uni-heidelberg.de/volltextserver/24850/`.
[8] J. Als-Nielsen and D. McMorrow *Elements of modern X-ray physics.* (John Wiley & Sons, Cambridge, England, 2011).
